# Prediction of bioconcentration factors in fish and invertebrates using machine learning

**DOI:** 10.1016/j.scitotenv.2018.08.122

**Published:** 2019-01-15

**Authors:** Thomas H. Miller, Matteo D. Gallidabino, James I. MacRae, Stewart F. Owen, Nicolas R. Bury, Leon P. Barron

**Affiliations:** a Department of Analytical, Environmental & Forensic Sciences, School of Population Health & Environmental Sciences, Faculty of Life Sciences and Medicine, King's College London, 150 Stamford Street, London SE1 9NH, UK; b Department of Applied Sciences, Northumbria University, Newcastle Upon Tyne NE1 8ST, UK; c Metabolomics Laboratory, The Francis Crick Institute, 1 Midland Road, London, NW1 1AT, UK; d AstraZeneca, Global Environment, Alderley Park, Macclesfield, Cheshire SK10 4TF, UK; e Division of Diabetes and Nutritional Sciences, Faculty of Life Sciences and Medicine, King's College London, Franklin Wilkins Building, 150 Stamford Street, London SE1 9NH, UK; f Faculty of Science, Health and Technology, University of Suffolk, James Hehir Building, University Avenue, Ipswich, Suffolk IP3 0FS, UK

**Keywords:** Modelling, PBT, Pharmaceutical, Bioconcentration, BCF, Machine learning

## Abstract

The application of machine learning has recently gained interest from ecotoxicological fields for its ability to model and predict chemical and/or biological processes, such as the prediction of bioconcentration. However, comparison of different models and the prediction of bioconcentration in invertebrates has not been previously evaluated. A comparison of 24 linear and machine learning models is presented herein for the prediction of bioconcentration in fish and important factors that influenced accumulation identified. R^2^ and root mean square error (RMSE) for the test data (n = 110 cases) ranged from 0.23–0.73 and 0.34–1.20, respectively. Model performance was critically assessed with neural networks and tree-based learners showing the best performance. An optimised 4-layer multi-layer perceptron (14 descriptors) was selected for further testing. The model was applied for cross-species prediction of bioconcentration in a freshwater invertebrate, *Gammarus pulex*. The model for *G. pulex* showed good performance with R^2^ of 0.99 and 0.93 for the verification and test data, respectively. Important molecular descriptors determined to influence bioconcentration were molecular mass (MW), octanol-water distribution coefficient (logD), topological polar surface area (TPSA) and number of nitrogen atoms (nN) among others. Modelling of hazard criteria such as PBT, showed potential to replace the need for animal testing. However, the use of machine learning models in the regulatory context has been minimal to date and is critically discussed herein. The movement away from experimental estimations of accumulation to in silico modelling would enable rapid prioritisation of contaminants that may pose a risk to environmental health and the food chain.

## Highlights

• Evaluation of 24 models to predict bioconcentration factors in fish is presented.
• Machine learning showed good predictive performance.
• First machine learning application to predict bioconcentration in invertebrates
• Cross-species modelling is limited by case similarity and biological variability.
• TPSA, LogD, and Mw were important descriptors for modelling accumulation processes.

## Graphical abstract

**Figure:**
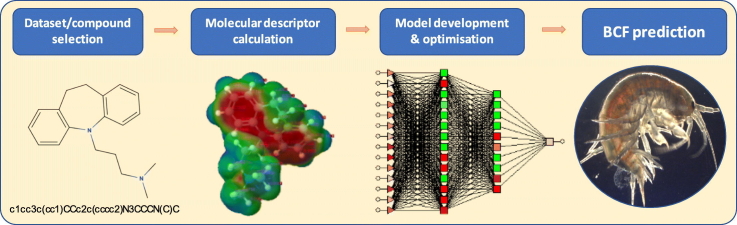

## 1 Introduction

Both terrestrial and aquatic environments experience pollution from a wide range of chemical contaminants. The presence of these contaminants is a cause for concern as they may elicit adverse effects to environmental and public health. Bioaccumulation of chemicals is critically important for understanding the risk of chemicals in the environment. The complexity of confounding factors that affect uptake make simple relationships that can confidently predict the accumulation elusive; but it may not have to be that way.

Live animal exposure studies are currently the norm, using many hundreds of fish for each assessment (Rovida and Hartung, 2009). Across the European Union (EU), various guidelines have been established for industry to minimise the risk posed by their chemical products. For pharmaceuticals in the EU this is regulated by the European Medicines Agency (EMA) and for other chemicals substances the regulations are outlined by the Registration, Evaluation, Authorisation and restriction of CHemicals (REACH) (European Commission, 2006; Euorpean Medicines Agency, 2006). According to REACH, any manufacturer of a chemical that exceeds quantities of 10 t per annum must submit a chemical safety assessment (CSA). For environmental risk assessment, part of the CSA includes persistence, bioaccumulation and toxicity (PBT) assessments. Alternatively, for pharmaceuticals environmental risk assessment (ERA) follows an initial screening (Phase I) where physico-chemical properties of the compound are determined (e.g. logP) and the expected exposure is estimated. The Phase I exposure estimation is calculated as the predicted environmental concentration (PEC). If the PEC is >0.01 μg L^−1^ then the pharmaceutical must undergo further testing to assess environmental fate and toxicity. However, it should be noted that substances with a logP >4.5, will trigger a PBT assessment (following REACH guidelines) regardless of the Phase I PEC.

For PBT assessments, existing available screening data and prior assessment information are used to determine whether a chemical is bioaccumulative (B) or very bioaccumulative (vB) by estimation of a bioconcentration factor (BCF) or bioaccumulation factor (BAF). Currently, pharmaceuticals are not restricted or replaced as would normally be defined under REACH. Furthermore, whilst PBT assessments are implemented, the persistence and bioaccumulation outcome of these assessments are not taken into consideration for authorisation purposes, as no legal provisions specifically cover persistent, bioaccumulative and toxic substances for pharmaceuticals (European Medicines Agency, 2016).

Laboratory testing for PBT brings with it a significant level of planning, quality control and cost (Rovida and Hartung, 2009). Therefore, *in silico* methodologies to predict BCF or BAF offers a potential advantage to more intelligently use data to characterise potential exposure and risk. Quantitative Structure Activity Relationships (QSARs) are becoming increasingly popular within ecotoxicological fields as they represent, perhaps, the only realistically feasible scenario to assess the environmental risk of the several thousand chemicals that are available on the market (Gissi et al., 2013). In addition, such models can be used to ethically reduce or replace animal testing and falls under the replacement, reduction and refinement (3Rs) framework (de Wolf et al., 2007). Further, effective *in silico* models could also be utilised to help shape future drugs in terms of ‘green by design’ ambitions (Lockwood and Saïdi, 2017).

More recently, more complex machine learning-based QSAR models involving artificial neural networks (ANNs), tree-based learners or support vector machines (SVMs) have been used to model BCF in fish (Fatemi et al., 2003; Lombardo et al., 2010; Zhao et al., 2008; Strempel et al., 2013). However, several variations of machine learning-type models exist and wider applications of such models for bioaccumulation prediction have not yet been evaluated to identify any added benefits. Furthermore, current QSAR models have only been applied to modelling fish bioaccumulation data and do not incorporate pharmaceutical data. The potential for application to other taxa such as invertebrates is also non-existent, mainly due to a shortage of available data.

The aim of this work was to develop and critically evaluate several machine learning-based modelling tools for prediction of bioconcentration factor (BCF) in both a fish (*Cyprinus carpio*) and an invertebrate species (*Gammarus pulex*) for the first time. An open access fish BCF dataset was used in the first instance to build and compare 24 different models for 352 different compounds. Subsequently, the best model was applied to both a set of fish and invertebrate BCF data to assess its potential for cross-species prediction. The invertebrate dataset also contained mainly pharmaceuticals. In parallel, independent models were developed ab initio on a smaller set of invertebrate BCF data alone to assess the degree of commonality with the model developed on fish BCF data. Finally, the importance of molecular descriptors to understand the potential for a chemical to accumulate in biota was assessed. The use of such rapid and flexible modelling approaches is now critical to support the 3Rs, aid greener design and to help meet the demand for PBT assessments of potentially large numbers of compounds, which could be expanded to new and emerging environmental contaminants across different species.

## 2 Materials and methods

### 2.1 Dataset generation and pre-processing

Bioconcentration factors were collated from the European Chemical Industry Council Long-range Research Initiative (Cefic LRI) project EC07 in collaboration with European Academy for Standardisation e.V (EURAS) which established the BCF gold standard database across multiple fish species and is freely available at http://ambit.sourceforge.net/euras/. BCFs were down-selected to reduce variability between different species and experimental conditions within the database. The BCF data used herein were specific to *C. carpio* and were included by the Chemicals Inspection and Testing Institute (Institute, 1992). Out of all BCF data, this sub-selection resulted in the largest dataset with a single fish species (n = 352) for modelling purposes. The reported BCFs represented whole-body values only and included pigments, pesticides, fungicides, herbicides, insecticides, polyaromatic hydrocarbons (PAHs) and polychlorinated biphenyls (PCBs), organochlorines, nitroaromatics, alkylphenols, aromatic hydrocarbons, organosulfurs and organotins. Approximately 36% of the dataset contained ionisable compounds (estimated from ACD labs, Percepta software). The invertebrate BCF dataset (n = 34) was collated from literature reported data (Ashauer et al., 2006; Ashauer et al., 2010; Meredith-Williams et al., 2012; Miller et al., 2017; Miller et al., 2016a) for the benthic freshwater organism, *G. pulex*. This species was selected as there was a relatively large amount of BCF data available when compared with other invertebrate species. For these, BCF data were only available for pharmaceuticals and pesticides and, again, represented whole-body values.

Simplified molecular input line entry system (SMILES) strings were generated for each compound using Chemspider (Royal Society of Chemistry, UK). Molecular descriptors were generated from SMILES strings using Parameter Client (Virtual Computational Chemistry Laboratory, Munich, Germany), and ACD Labs Percepta (Advanced Chemistry Development Laboratories, ON, Canada). Approximately 450 descriptors were initially generated covering constitutional, topological, geometrical and physico-chemical properties. The fish and invertebrate datasets were pre-processed to remove any zero variance descriptors or descriptors that were erroneous. All BCF data used for modelling was log transformed for improved predictive accuracy.

### 2.2 Feature selection

Descriptors were down-selected using three different feature selection algorithms, the first of which was a genetic algorithm (GA). The GA parameters were set to population = 500, generations = 250, mutation rate = 0.1 and cross-over rate = 1. The remaining two selection methods were part of stepwise regression which included a forward selection algorithm (FA) and backwards selection algorithm (BA). The feature selection algorithms used a generalised regression neural networks (GRNN) to monitor the error associated with the selected descriptors, where descriptor sets were optimised when the error showed no improvement. The use of GRNN for descriptor selection is very fast and requires minimal processing power. The performance of each feature selection algorithm was characterised by then testing several thousand neural networks and evaluating the predictive performance of the models based on the error of the predictions. The best feature selection method was the GA, which resulted in the down-selection of descriptors to a total of 14 that included 6 topological descriptors; radial centric information index (ICR), Narumi harmonic topological function (Hnar), ramification index (Ram), superpendentic index (SPI), spanning tree number (STN), topological polar surface area (TPSA), 4 constitutional descriptors; number of hydrogens (nH), number of carbons (nC), number of nitrogens (nN), molecular weight (MW), 3 electrotopological descriptors; maximal electrotopological negative variation (MAXDN), maximal electrotopological positive variation (MAXDP), mean atomic Sanderson electronegativity (Me) and 1 physico-chemical property; the octanol-water distribution coefficient (logD) (See SI, Table S3).

### 2.3 Modelling approaches

Two different software packages were used to assess the applicability of several in silico models in predicting bioconcentration. Trajan 6.0 (Trajan Software Ltd., Lincolnshire, UK) was used to build and evaluate artificial neural networks. In addition, this software was also used for the feature selection and the same descriptors were used in both modelling software packages. Models developed and optimised in Trajan included generalised regression neural networks (GRNN), radial basis function networks (RBF) and 3−/4-layer multilayer perceptrons (MLP). Training of the MLPs used two training algorithms referred to as back propagation (BP) and conjugate gradient descent (CGD), models were trained for 100 iterations. The optimised model was a four-layer MLP. The first and fourth layers were the inputs (molecular descriptors) and outputs (logBCF), respectively. The second and third layers (hidden layers) contained 14 and 10 nodes, respectively. Regularisation was performed with the use of early stopping to prevent over-training of the dataset. Parameter tuning was performed by changing the number of hidden layers and nodes and assessing the model performance on the verification and test subsets. The subsets of cases presented to the neural networks were split so that 242 compounds (70%) were used for training, 55 compounds (15%) for verification and 55 compounds (15%) for testing the networks. Normalisation of the input features showed no improvement in performance of the networks and training was performed without centred or scaled descriptors.

In the second software package, modelling was performed using the R statistical computing language (freely available from https://www.r-project.org). Here, 19 predictive models from different kinds of learner categories including both linear and non-linear models were trained and tested. These included, ordinary least-squares regression (OLM, package: *stats*), partial least-squares (PLS, package: *pls*), ridge regression (RR, package: *elasticnet*), elastic net (EN, package: *elasticnet*), quantile regression with LASSO penalty (QRL, package: *rqPen*) multivariate adaptive regression splines (MARS & B-MARS, package: *earth*), k-nearest neighbours regression (KNN, package: *caret*), extreme learning machines (ELM, package: *elmNN*), support vector machines with radial basis function (SVM-R, package: *kernlab*) and polynomial (SVM—P, package: *kernlab*) kernels, random forest exploiting classification and regression trees (RF-CART, package: *randomForest*) and conditional inference trees (RF-CIT, package: *party*) algorithms as base learners, boosted trees (BT, package: *gbm*) and Cubist regression (CR, package: *Cubist*). MLPs (3–5 layers) with 1 hidden layer (ANN-1HL, package: *nnet*), averaged 1 hidden layer (ANN-a1HL, package: *nnet*), 2 hidden layers (ANN-2HL, package: *RSNNS*) and 3 hidden layers (ANN-3HL, package: *RSNNS*) were also tested. For this modelling approach, the same molecular descriptors and logBCF were used again as input and output variables. The dataset was split into two subsets, training data (70%) and test data (30%). Normalisation of the data was required for the modelling application and the dataset was both centred and scaled. Parameter tuning was performed by resampling of the training subset following a 10-fold cross-validation scheme repeated five times and implemented through the *caret* package. Performance of each model was assessed from the root-mean square error (RMSE) and the correlation coefficient (R^2^). The best model for each regression method was then selected, retrained on the entire training dataset and used to predict cases in the test dataset. Final datasets used for modelling the optimised models are given in the SI (Table S1 & S2). The finalised models were all tested according to OECD guidelines (OECD, 2007) for QSAR model validation.

## 3 Results and discussion

### 3.1 Down-selection of input features for modelling BCFs in fish

The down-selection of the input features was assessed using three different feature-selection algorithms. Stepwise methods that included forwards or backwards selection (FA/BA) reduced the number of descriptors from 180 down to 72, whilst the GA reduced the number of descriptors to 66. The GA showed better correlation between selected descriptors with logBCF compared to stepwise algorithms (Fig. S1). For both BA and FA, the selection process converged to the same local minima indicating that there was no difference in using either algorithm. The improved performance of the GA is due to selection of descriptors from multiple points in the descriptor space, as opposed to FA or BA that start selection from a single point. Thus, approaching global minima is more likely to arise when using the GA over stepwise selection methods.

From the 66 descriptors selected by the GA, the top 22 descriptors plus an additional two user curated descriptors were selected for further modelling (See SI, Table S3). These additional descriptors were logD and number of hydrogen acceptor groups (nHAcc) and were chosen for their previously demonstrated influence on accumulation in biota (Palm et al., 1997; Kah and Brown, 2008). All descriptors were then tested across several thousand MLPs (three and four-layer) where the Trajan software sub-selected the best from the group of 24 descriptors based on model performance (MLPs yielded the best performance over other model types in terms of R^2^ and RMSE). The descriptors were down-selected to a total of 14 that showed relatively good performance across MLPs tested and were subsequently used in both modelling approaches discussed herein (Table S3). Given the scale of BCF data used for training (n = 242), the 5:1 Topliss threshold set out by the OECD guidelines (OECD, 2007) for the ratio of numbers of cases to descriptors was acceptable at 17:1.

### 3.2 Comparison of model performances for prediction of fish BCFs

The results of both modelling approaches are shown in Table 1. For models trained in R, the highest RMSE values were observed for OLM (1.203), followed by PLS (1.164) and then QRL (1.112). The relatively poor performance of such linear models may be expected as modelling such a biologically complex process is not likely to follow linear relationships using simple molecular descriptors. Even with well-studied descriptors, such as logP, there is a non-linear trend with accumulation over a specific threshold (generally, logP >6) (Devillers et al., 1998). However, when used as a sole descriptor, logP may exclude processes that are also important for accumulation. For example, elimination and metabolism rates may impact net accumulation as well as more specific physiology such as carrier mediated transport and protein binding (Dobson and Kell, 2008) will also influence accumulation, especially for emerging contaminant classes such as pharmaceuticals. By comparison, better performance was achieved using higher complexity models. The lowest RMSEs were observed for RF-CART (0.771), followed by BT (0.789) and RF-CIT (0.821), i.e. three tree-based machine learners. Next, ANNs and SVMs performed very similarly to tree learners, e.g. SVM-R (0.841), ANN-a1HL (0.859) and ANN-3HL (0.880).

**Table 1:** Comparison of model performance for the prediction of BCF in *Cyprinus carpio*. MAE is the mean absolute error and NA indicates the metric was not applicable.

	Model	RMSE			R^2^			MAE		
		Training	Verification	Test	Training	Verification	Test	Training	Verification	Test
Trajan	*Linear*	0.785	1.052	0.832	0.532	0.390	0.521	0.619	0.835	0.608
	*GRNN*	0.830	0.893	0.873	0.673	0.400	0.569	0.664	0.893	0.718
	*RBF*	0.723	0.689	0.584	0.651	0.635	0.725	0.565	1.600	0.450
	*3-MLP*	0.689	0.538	0.337	0.675	0.770	0.659	0.548	1.608	0.553
	*4-MLP*	0.403	0.524	0.644	0.887	0.819	0.702	0.313	0.380	0.530
	Model	Training	Cross-Validation	Test	Training	Cross-Validation	Test	Training	Cross-Validation	Test
R	*OLM*	0.719	0.771	1.203	0.621	0.570	0.234	0.560	NA	0.778
	*PLS*	0.722	0.769	1.164	0.618	0.571	0.254	0.564	NA	0.765
	*RR*	0.725	0.766	1.083	0.614	0.576	0.304	0.568	NA	0.753
	*EN*	0.729	0.760	1.054	0.612	0.582	0.314	0.577	NA	0.754
	*QRL*	0.733	0.757	1.112	0.607	0.585	0.284	0.562	NA	0.770
	*KNN*	0.517	0.683	0.902	0.807	0.665	0.468	0.404	NA	0.648
	*ELM*	0.673	0.756	1.014	0.668	0.593	0.346	0.529	NA	0.768
	*ANN-1HL*	0.596	0.751	0.877	0.739	0.597	0.505	0.462	NA	0.620
	*ANN-a1HL*	0.395	0.672	0.859	0.888	0.678	0.518	0.319	NA	0.612
	*ANN-2HL*	0.232	0.834	1.022	0.962	0.560	0.370	0.174	NA	0.680
	*ANN-3HL*	0.454	0.795	0.880	0.860	0.582	0.520	0.345	NA	0.624
	*MARS*	0.539	0.730	1.014	0.787	0.632	0.390	0.425	NA	0.696
	*B-MARS*	0.500	0.681	0.899	0.819	0.673	0.479	0.395	NA	0.633
	*SVM-R*	0.383	0.644	0.841	0.893	0.704	0.537	0.261	NA	0.590
	*SVM-P*	0.699	0.747	1.029	0.643	0.594	0.340	0.539	NA	0.729
	*RF-CART*	0.292	0.675	0.771	0.956	0.688	0.633	0.231	NA	0.589
	*RF-CIT*	0.605	0.739	0.821	0.762	0.630	0.586	0.485	NA	0.652
	*BT*	0.249	0.660	0.789	0.957	0.687	0.593	0.187	NA	0.587
	*CR*	0.353	0.678	0.973	0.910	0.673	0.431	0.282	NA	0.628

Models tested in Trajan showed particularly good performance, in comparison to those built in R. The lowest RMSE value was observed for a 4-layer MLP (0.524), followed by 3-layer MLP (0.538), RBF (0.689), GRNN (0.893) and Linear (1.052). In absolute terms, definitive conclusions cannot be drawn from direct comparison of modelling approaches (i.e., Trajan vs. R), as tuning and training methods between modelling software packages are slightly different. However, overall results converged to support the higher reliability of non-linear approaches for modelling logBCF from molecular descriptors.

Model complexity does not necessarily mean better predictive performance by default, as several non-linear machine learners did not perform well at all. These included ELM and SVM—P, where the RMSE values observed on the test set were > 1. Although ELM is a feedforward neural network, the weights associated with the neurons in the network are not updated and thus the initialisation of the network is a random selection of weights that may not model the output reliably. The EN outperformed QRL and RR models, where the EN is a combination of the penalties (L1 and L2 regularisation) used by both models that usually leads to better predictive performance. The RR model RMSE for the test set data was also lower than the RMSE for the QRL model. This can be observed when comparing RR and QRL methods, as the penalty associated with LASSO can lead to the omission of highly correlated covariables and thus lead to lower model robustness.

Limitations of predictive performance may also stem from the raw data. For example, the dataset used herein did not report individual experimental pH, but instead reported a range from 6.0 to 8.5. Therefore, descriptors such as logD that require pH data may become limited and especially where molecular pK_a_ lies within this 2.5 pH unit range. LogD has been shown in several works to influence uptake and accumulation (Nakamura et al., 2008; Rendal et al., 2011; Karlsson et al., 2017). As a compromise, we calculated logD at pH 7, but this may have been different to the exact experimental pH and may have added to predictive inaccuracy across the whole analyte set. Lastly, it is also likely that BCF/BAF prediction will be influenced by variance in biotic factors such as ventilation rates, age, genetic factors and metabolism and lay beyond our ability to determine in more detail (Mackay and Fraser, 2000; Rubach et al., 2010a).

**Fig. 1:**
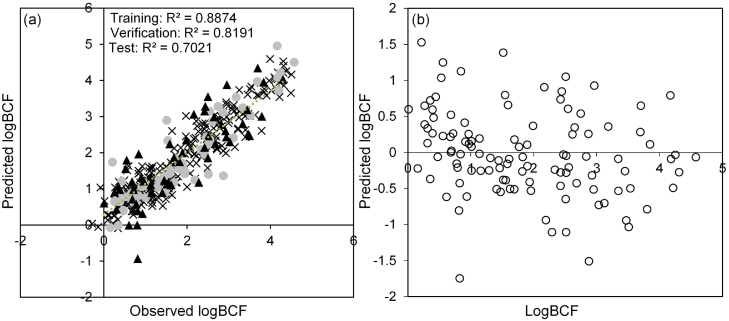
(a) linear regression of the predicted logBCF values versus the observed logBCF values in fish using the 4-MLP developed in approach 1, training data (crosses, n = 242), verification data (circles, n = 55) and test data (triangles, n = 55). (b) Raw residuals of the predicted logBCF data in fish for the verification and test data only.

MLP models trained in Trajan offered the best performance. Consequently, this model was chosen for further investigation in line with the OECD validation guidelines to assess validity of QSAR modelling (Fig. 1). The mean absolute error (MAE) corresponded to 0.38 logBCF units for the verification subset (internal validation set) and 0.53 logBCF for the test subset (external validation set), as shown in Table 1. The RMSE for verification and test subsets were 0.524 and 0.644, respectively. The predictive performance of this model was better or comparable to all models in the literature that have attempted to model accumulation processes. Dearden and Shinnawei (Dearden and Shinnawei, 2004) used a linear QSAR approach to predict BCFs for 135 chemicals with an *R*^*2*^ of 0.637 and RMSE of 0.661 logBCF units. Another QSAR model by Sahu and Singh (Sahu and Singh, 2009) used multiple linear regression to predict BCFs for 131 organic compounds with a RMSE of 0.556 log units. However, this model was not validated against a test subset and therefore generalised applicability of the model performance is arguably limited.

In alternative approaches to linear QSAR models, other machine learning approaches have also been reported (Fatemi et al., 2003; Lombardo et al., 2010; Zhao et al., 2008). A MLP predicted BCFs for 9 test compounds with an average absolute error of 0.33 ± 0.22 log units (Fatemi et al., 2003). Whilst the errors were low, too few compounds were tested to provide a reliable assessment of its generalisability. In another approach, Zhao et al., (Zhao et al., 2008) used SVM, RBF and MLR models individually. Better performance was observed when two RBF models (using different descriptors) were combined into a ‘hybrid’ model to predict logBCF. The developed model showed an *R*^*2*^ of 0.6917 for an external test set with a reported RMSE of 0.69 logBCF units for 119 compounds showing similar performance to the fish-based MLP presented here, using a single MLP. The hybrid model also showed a limitation in the training set, where several cases were not modelled correctly between the ranges of logBCF 4 to 5 and was observed by a plateau in the regression analysis.

### 3.3 A remark on outliers and the applicability domain

**Fig. 2:**
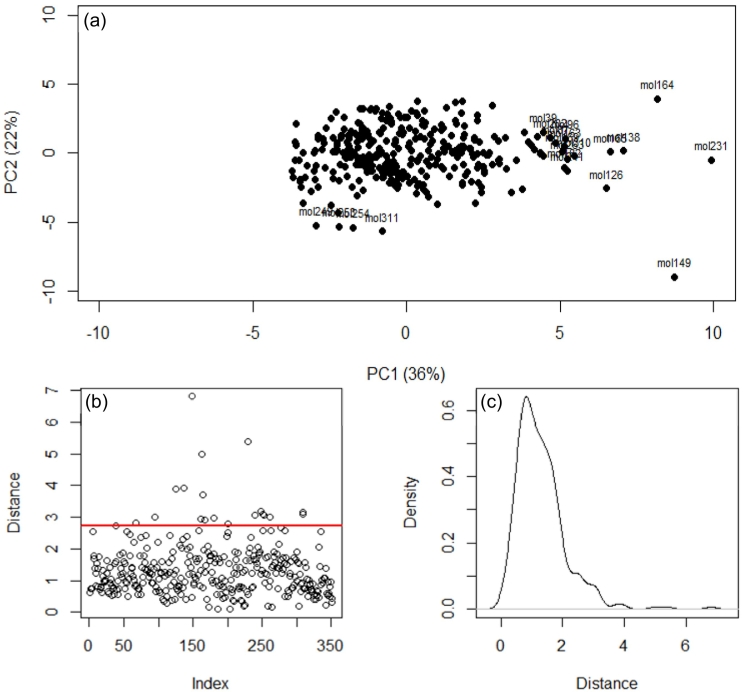
(a) Principal component analysis used for visualisation of the case similarity based on the 14 modelled descriptors (i.e. applicability domain). (b) Distances between cases in the PCA space with a threshold applied (0.975 quantile of χ^2^ distribution) designated by the red line (c) the distribution of cases based on distance in the PCA space.

Training and testing of all models led to the observation of several common outliers. The reason for poor prediction for such cases may stem from under representation in the dataset used for modelling. The spread of input and output data between training and validation subsets showed that there was no significant difference between the spread or skew of the data (Fig. S2). However, using PCA analysis and distances between the descriptor spaces there were several cases that did not cluster well with the remaining data (Fig. 2a). For example, logBCF for perfluorotributylamine was predicted poorly across the majority of trained models. The use of PCA and descriptor data spacing in this way enabled characterisation of the applicability domain (AD) for a given model. A threshold may then be used to determine cases that fall outside the domain and are likely to have higher predictive error (Fig. 2b) (Aalizadeh et al., 2016; Weaver and Gleeson, 2008).

According to the OECD QSAR model validation guidance (OECD, 2007), consideration of models for regulatory purposes must be associated with a defined domain of applicability under Principle 3. However, one key consideration in the use of distance-based ADs is that input descriptors are not used equally by the model (Netzeva et al., 2005). Therefore, such ADs may not accurately identify those cases having a greater predictive error in every case. This was observed for outliers in the PCA analysis, but where logBCF was predicted relatively well and vice versa. For example, di-2-naphthyldisulfide was not an outlier in the AD but was poorly predicted across all models. On the other hand, pigment yellow-12 was an AD outlier, but logBCF was predicted well by the majority of models.

Poor predictive accuracy for molecularly similar compounds could be also caused by other factors such as poor quality raw data or too few representative training cases for the model to learn from. It has been shown previously that experimental BCF data can vary from 0.42 to 0.75 log units (Lombardo et al., 2010; Dimitrov et al., 2005; Arnot and Gobas, 2006). Nevertheless, even with the limitations associated with defining an AD, it is useful and important to identify any cases that might not be reliably predicted so that rapid prioritisation of compounds can begin. Only for these cases, may it then be appropriate to revert to experimental testing.

### 3.4 Machine learning in a regulatory context

Several of the developed machine learning tools in Table 1 showed potential for the replacement and reduction in animal use. However, it is important to recognise the complexities of machine learning approaches from the outset, especially where they are intended for use in regulation. Under Principle 2 of the OECD guidelines, models used in this way must be based on “unambiguous algorithms”. In particular, it is highlighted that two significant limitations exist regarding artificial neural networks, for example. These are: (a) the necessity for large (BCF) datasets to develop suitable models (which do not exist for some classes of compounds, like pharmaceuticals) and also (b) that these types of machine learning tools are more ambiguous than other types of model, especially those that are linear in nature. For the latter, the guidance is vague concerning appropriateness of ANNs for use under this specific principle but infers that it is an acceptable limitation. Furthermore, the definition of an unambiguous algorithm is in fact ambiguous and should be further refined to prevent confusion to the reader. This principle could be applied in different ways to different models and may cover the generation of molecular descriptors, the feature selection algorithms used, the learning process (for machine learners where the ambiguity lies) and the final model (Gramatica, 2007). The majority of the literature seems to have focused on linear models perhaps as a result, mainly to aid in mechanistic understanding and to allow expert interpretation of individual chemicals to provide extra assurance in predicted data (linked to Principle 5).

Principle 5 of the OECD guidelines relates to mechanistic interpretability of QSAR models (if possible). This can be considered a limitation for machine learning algorithms if the aim is to achieve an interpretable model, such as would normally be expected of linear models such as OLS or PLS regression. The OECD guidelines also remain vague regarding mechanistic interpretation of machine learners. However, whist linear relationships may not be apparent, descriptor sensitivity analyses can indicate the importance of individual descriptors and thus enables interpretation of factors that influence the modelled process. Bioconcentration processes are not simple and extensive datasets are extremely impractical to curate experimentally. Therefore, complex non-linear models may provide a more rapid solution to regulatory decision-making meantime. Therefore, we suggest that guidelines for QSAR model validation need to be expanded to better define the scope of applicability of all the different types of machine learning tools and their fitness for purpose in a regulatory context.

For PBT testing, the same regulations are triggered when a threshold for bioaccumulation is reached, regardless of the extent to which the threshold is exceeded. Thus, if the value is classified within the correct category of non-bioaccumualtive (nB), bioaccumulative (B) or very bioaccumulative (vB), the model will be useful in the context of PBT assessments. Variability in measurement can arise from kinetic modelling approaches (Miller et al., 2016a), biological/physiological variability (age, health, lipid content etc.) (Rubach et al., 2010a; Verhaar et al., 1999; Hendriks et al., 2001; Buchwalter et al., 2002; Rubach et al., 2010b) and experimental conditions (pH, temperature, etc.) (Nakamura et al., 2008; Karara and Hayton, 1989). As such, reported BCFs have been shown to differ by 1–2 orders of magnitude even within the same species (Rubach et al., 2010a).

The 4-layer MLP here showed a correct classification rate of 90% across the verification and test subsets. The 10% misclassification of cases was split to 6% of cases predicted as false negatives and 4% of cases predicted as false positives (See SI, Fig. S3). This is consistent with the hybrid model developed by Zhao et al. which has shown classification accuracies ranging from 91% to 98% (Lombardo et al., 2010; Zhao et al., 2008). It is possible that using QSARs for classification instead of regression analysis may improve the accuracy and without the need for the application of a bias. This would be particularly suitable for bioaccumulation assessments where only a threshold value determines the level of regulation enforced.

Some studies have reported the application of models for classification of bioaccumulation thresholds, with accuracies ranging from 84.5–91.1% (depending on model type) (Sun et al., 2008) and 91.7% (Strempel et al., 2013). The authors that used tree-based learners also used these models for quantitative prediction achieving RMSE of 0.554 and R^2^ of 0.836 on the test set data (Strempel et al., 2013). The models tested across the literature have tended to achieve similar performance for both classification and prediction. The agreement in performance between different works and the comprehensive model evaluation here, support that in silico methods should be adopted for chemicals where environmental uptake data are limited to enable flexible, cheap and rapid PBT assessment for compound prioritisation. Furthermore, it suggests that the use of chemical descriptors may only be able to achieve a certain level of predictive or classification performance for modelling approaches where other variables become important as mentioned above.

### 3.5 Can the developed model be used for cross phylum prediction?

**Fig. 3:**
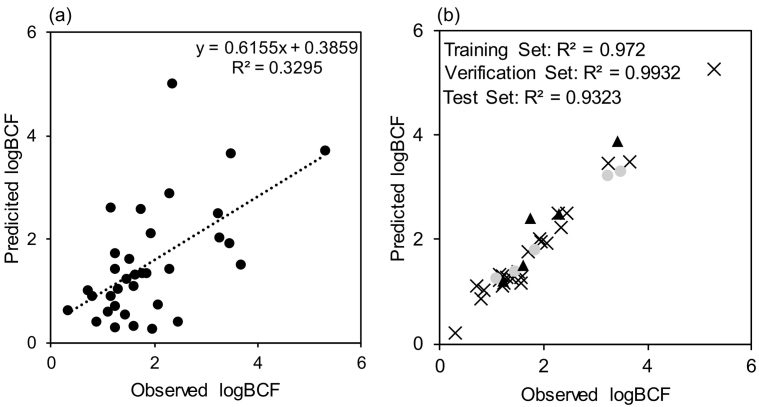
(a) Comparison of the predicted logBCF data versus the observed logBCF in invertebrates using the fish-based 4-layer MLP. (b) Regression of a separately developed and optimised model trained with the invertebrate BCF data (*Gammarus pulex*), training set (crosses, n = 24), verification set (circles, n = 5) and test set (triangles, n = 5).

There is little understanding of whether accumulation will be similar across the invertebrate phylum. The dominant site of uptake for waterborne micropollutants in fish is across the gills and therefore accumulation across taxa may be significantly different for differing modes of respiration. Other factors such as size, enzyme speciation and lipid content may also influence the accumulation potential (Rubach et al., 2010a). The optimised model for fish was applied to the prediction of logBCF in a freshwater invertebrate, *Gammarus pulex* (Fig. 3a). The accumulation data in *G. pulex* predominantly covered pharmaceuticals and pesticides. The fish-based MLP showed relatively low predictive performance for the invertebrate accumulation factors. The correlation between observed and predicted BCF was *R*^2^ 0.3295 with a MAE of 0.80 ± 0.65 log units, which indicated that the model generalisations between species were limited. The largest predictive error was for the compound imipramine that was overestimated by 2.7 logBCF units. This compound in a previous study had considerable variation in the estimated BCF (212–4533) depending on the method of estimation used (Miller et al., 2016a).

A significant difference in BCFs between trophic levels has been shown with higher trophic levels displaying increased BCFs (LeBlanc, 1995). This trend would suggest that the BCF predictions of the invertebrates might be overestimated but the opposite was observed (62% of cases were underestimated). In addition to the biological complexity between species, another confounding factor to affect the predictive accuracy and generalisability is the compound class. The fish model included no pharmaceutical compounds whereas the invertebrate BCF data contained 18 cases (~53%). Inspection of the molecular similarity between the datasets indicated that the invertebrate and fish datasets were dissimilar (Fig. S4). Thus, the bioconcentration potential may not follow the same relationships with neutral hydrophobic organic contaminants.

The fish-based model was subsequently reinitialised and trained on the invertebrate dataset only (using the same descriptors) (Fig. 3b). The invertebrate model showed good correlation with *R*^2^ of 0.9605 with 0.972 for the training set, 0.9932 for the verification set and 0.9323 for the test set. The model demonstrated good accuracy across the verification and test subset with a MAE of 0.07 ± 0.08 logBCF units for the verification set and 0.29 ± 0.27 logBCF units for the test set. The successful retraining of the model to invertebrate data suggests that case representation (i.e. compound class) is likely to limit models that are applied across taxa. An alternative approach to overcome this could involve development of a model with two or more outputs to represent different species, but commonality in BCF cases would be required for both species. Whilst the predictive accuracy of the retrained model was very good, it is also limited by the small number of cases used. Generalisability is also likely to be limited given the ratio of cases to descriptors (Topliss ratio of ~2.5:1) Nevertheless, and as new BCF data emerges, this approach holds excellent potential by using the same molecular descriptors for BCF predictions in two very different species. In addition, to using the fish-based model to predict invertebrate BCFs we also used the invertebrate-based model to predict fish BCFs of pharmaceuticals reported in the literature (Fig. S5). The invertebrate model was able to predict BCFs within the reported range for 45% of the compounds selected (n = 11). The remaining compounds, with the exception of sertraline and gemfibrozil, were predicted relatively well even though they were not within the reported ranges. Sertraline is an interesting case as although it has not shown very high bioconcentration in fish (BCFs: <1–626) (Grabicova et al., 2014; Lajeunesse et al., 2011; Tanoue et al., 2014; Togunde et al., 2012; Xie et al., 2015) there have been reported BCF values of up to 32,022 in invertebrates (namely, *Lasmigona costata* (de Solla et al., 2016) and 990 in *Planorbid sp.* (Du et al., 2015)). As the model used here was trained on BCFs from an invertebrate species, it may not correlate well with fish BCF data, suggesting that cross-phylum predictive modelling may be limited by both case representation and biological variation. However, as the models here used the same descriptors this enables flexibility in retraining optimised models and inevitably as more BCF data is generated for the same compounds in different species, this technology could be used to map accumulation across taxa more effectively. It is critically important to understand uptake (internal concentration) across taxa as the conservation of pharmaceutical targets extends widely (Verbruggen et al., 2018).

### 3.6 Model sensitivity to descriptors: interpreting accumulation through chemistry

**Fig. 4:**
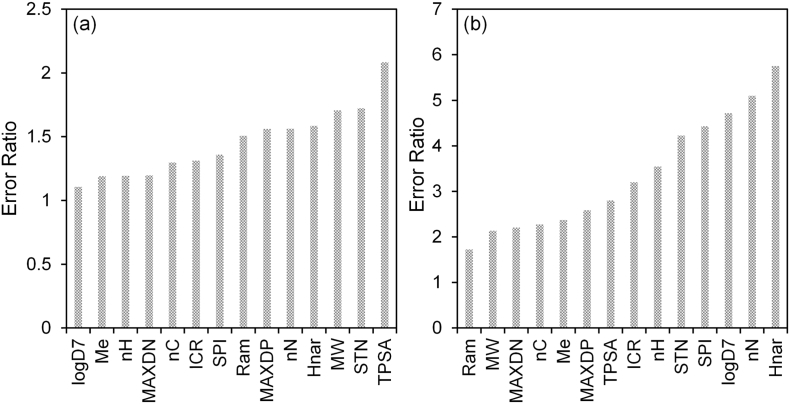
Descriptors sensitivity analysis performed by removing a descriptor from the model and assessing the affected performance. Increased error ratios indicate more important descriptors. (a) descriptor sensitivity for the fish-based model and (b) for the invertebrate-based model.

Whilst machine learning models are more difficult to interpret due to the non-linear functionality, collinearity and/or curvilinearity; the importance of the 14 descriptors described here still offered some mechanistic understanding of the processes involved (Fig. 4). For the fish-based model, the most important descriptor was TPSA with an error ratio of 2.08. Higher error ratios correspond to increased predictive error for all compounds upon removal of this descriptor from the dataset. Previous investigations have demonstrated that descriptors related to polarisability, hydrophobicity and hydrogen bonding of the molecule is important to modelling BCFs (Zhao et al., 2008; Dearden and Shinnawei, 2004; Gramatica and Papa, 2003). TPSA is defined as the surface area occupied by nitrogen and oxygen atoms including connected hydrogen atoms (Pajouhesh and Lenz, 2005). Polar surface area has also been shown to influence drug absorption in humans, where increasing polar surface area decreases the drug fraction absorbed (Palm et al., 1997; Kelder et al., 1999). The relationship between bioconcentration and TPSA may be dependent on several factors such as permeation through the lipid bilayer, binding of polar functional groups to epithelial membranes and the size of hydration shell around a molecule (Skyner et al., 2015).

Permeation through cellular membranes was further supported by the importance of MW to the model. The size of a molecule also affects permeation and diffusion through membranes (Lipinski's rule of five (Tice, 2001)). It has previously been demonstrated that dye pigments did not show bioaccumulation in fish due to their large molecular size (Anliker et al., 1988a). In another study, it was suggested that there is a threshold diameter value of 1.5 nm which governed bioconcentration in addition to hydrophobicity (Dimitrov et al., 2002). Strempel et al., (Strempel et al., 2013) also found that molecular weight, molecular diameter, TPSA and logD were important for classification and prediction of bioaccumulation.

Topological descriptors such as STN, Hnar, Ram, SPI and ICR were also found to be important. These indices are useful especially for differentiating constitutional isomers (except enantiomers) (Randić et al., 1988). Error ratios for STN, Hnar, ICR, SPI and Ram spanned from 1.31–1.72. These indices are related to molecular branching/shape and the importance of these descriptors relate to molecular size which can influence bioconcentration (Anliker et al., 1988b; Opperhulzen et al., 1985). MAXDN and MAXDP relate to the partial charges on atoms relative to their topological position within the molecule and therefore relate to the nucleophilicity and electrophilicity of a molecule (Gramatica et al., 2000). Aside from polarity-related accumulation across cellular membranes, it is also possible that these are associated with metabolic activity (from nucleophilic or electrophilic attack). The importance of other electrotopological descriptors (along with molecular flexibility) has been previously shown for modelling bioconcentration (Wang et al., 2008).

Interpretation of the relative importance of descriptors is affected by collinearity or multicollinearity (See SI, Table S4 & S5). The collinearity of the descriptors showed that molecular weight was collinear with SPI (R = 0.794) and Ram (R = 0.696). The descriptor Ram was also collinear with SPI (R = 0.787) and STN was collinear with HNar (R = 0.748). The relation between these topological descriptors and molecular weight is that they all describe molecular size (shape, volume, weight) to some extent. Therefore, the rank importance of these particular descriptors should be approached with some caution. Whilst the error ratio is higher for certain descriptors that are collinear, their removal from the network model may not correctly determine the ratio value due to redundant information. Nevertheless, the descriptor sensitivity can still be useful for directing mechanistic and experimental studies. This was shown recently in a neural network application to passive sampling (Miller et al., 2016b) which was later followed by a mechanistic study (Morin et al., 2018), that supported the interpretation of the model.

The invertebrate-based MLP used the same descriptors as the fish-based model, but the network was reinitialised and retrained. The retraining of the network also showed that the importance of the descriptors changed from the fish-based model. The most important descriptor was HNar (error ratio = 5.75) followed by nN (error ratio = 5.09) and logD (error ratio = 4.71). The increased importance of the number of nitrogen atoms likely reflected the number of pharmaceutical compounds in the dataset. In addition, logD increased in rank to the top three descriptors in the invertebrate model. The increased sensitivity of the model to logD also relates to training of the model with ionisable pharmaceuticals and is in agreement with other studies showing logD to be important in accumulative processes (Strempel et al., 2013; Morin et al., 2018). Whilst hydrophobicity may be a principal factor of bioconcentration, it is possible that carrier-mediated transport may also play an important role. Both models here demonstrated that other variables also strongly influence BCF prediction. Thus, QSAR models that rely solely on logP or logD in our opinion are limited in their application.

It is important to consider that descriptors not used in this work may also have a potential for BCF modelling. For example, the major mechanism of transport across epithelia tissue is passive diffusion and so it is also possible that diffusion coefficients could potentially be an important descriptor for consideration among others, however these descriptors are difficult to acquire and therefore reduce the practicability of a model based on these.

## 4 Conclusions

The work presented herein has shown that in silico modelling approaches are a powerful approach to predict bioconcentration of environmental contaminants, enabling rapid prioritisation of compounds during ERA. The approach could be used to better understand bioaccumulation, and the molecular descriptors that drive it; moving the science beyond simple hydrophobicity models that poorly account for the complexity of pharmaceuticals. Cross-species prediction of accumulation warrants further investigation as the results indicate both case representation and biological variability might limit prediction of accumulation between different taxonomic groups. Nevertheless, the use of machine learning has been increasing within the field and is necessary to improve our understanding of biological processes that affect environmental health. The interpretation of descriptors here is critical as it demonstrates that, in addition to rapid prediction of bioconcentration factors, *in silico* models are useful for mechanistic understanding which in turn can be used to direct further work. This is particularly true for pharmaceutical uptake in biota, where the mechanisms that govern uptake, elimination and accumulation processes are still not fully understood. Excellent potential exists for rapid screening using machine learning technology in future ERA, without the need for costly and ethically challenging animal experiments. Finally, the OECD QSAR validation guidelines for machine learners are inexplicit and we suggest these guidelines should be expanded with more focus on this type of modelling approach. This will begin to address the applicability and usefulness of these models for regulatory schemes such as REACH where PBT assessments are required for several thousand chemicals.

## Appendix A Supplementary data

Supplementary tables Image 1

Supplementary figures Image 2
